# MAPK1^E322K^ mutation increases head and neck squamous cell carcinoma sensitivity to erlotinib through enhanced secretion of amphiregulin

**DOI:** 10.18632/oncotarget.8188

**Published:** 2016-03-18

**Authors:** Yihui Wen, Hua Li, Yan Zeng, Weiping Wen, Kelsey P. Pendleton, Vivian W.Y. Lui, Ann Marie Egloff, Jennifer R. Grandis

**Affiliations:** ^1^ Department of Otolaryngology, The First Affiliated Hospital of Sun Yat-sen University, Sun Yat-sen University, Guangzhou, China; ^2^ Department of Otolaryngology, University of Pittsburgh School of Medicine, Pittsburgh, Pennsylvania, USA; ^3^ Department of Otolaryngology Head and Neck Surgery, University of California at San Francisco, San Francisco, California, USA; ^4^ Department of Pharmacology and Pharmacy, School of Biomedical Sciences, Li-Ka Shing Faculty of Medicine, The University of Hong Kong, Pok Fu Lam, Hong Kong SAR; ^5^ Departments of Molecular and Cell Biology and Otolaryngology, Boston University, Boston, Massachusetts, USA; ^6^ Clinical and Translational Science Institute, University of California at San Francisco, San Francisco, California, USA

**Keywords:** head and neck cancer, MAPK1, ERK2, mutation, amphiregulin

## Abstract

Epidermal growth factor receptor (EGFR) tyrosine kinase inhibitors (TKIs) have not been effective in unselected head and neck squamous cell carcinoma (HNSCC) populations. We previously reported an exceptional response to a brief course of erlotinib in a patient with advanced HNSCC whose tumor harbored a *MAPK1*^E322K^ somatic mutation. *MAPK1*^E322K^was associated with increased p-EGFR, increased EGFR downstream signaling and increased sensitivity to erlotinib. In this study, we investigated the mechanism of *MAPK1*^E322K^-mediated EGFR activation in the context of erlotinib sensitivity. We demonstrated increased AREG secretion in HNSCC cell lines harboring endogenous or exogenous MAPK1^E322K^ compared to wild type *MAPK1*. We found inhibition or knockdown of MAPK1 with siRNA resulted in reduced secretion of AREG and decreased sensitivity to erlotinib in the setting of *MAPK1*^E322K^. MAPK1^E322K^ was associated with increased AREG secretion leading to an autocrine feedback loop involving AREG, EGFR and downstream signaling. Knockdown of AREG in HNSCC cells harboring *MAPK1*^E322K^ abrogated EGFR signaling and decreased sensitivity to erlotinib *in vitro* and *in vivo*. These cumulative findings implicate increased AREG secretion and EGFR activation as contributing to increased erlotinib sensitivity in *MAPK1*^E322K^ HNSCC.

## INTRODUCTION

Clinical responses to epidermal growth factor receptor (EGFR) tyrosine kinase inhibitors (TKIs) in solid tumors lacking activating EGFR mutations are rare [1-3]. A complete “exceptional” response to EGFR TKI when *EGFR* is wild type suggests that there are additional genetic contributors that mediate EGFR TKI sensitivity [4, 5]. We recently reported the case of a patient with locally advanced head and neck squamous cell carcinoma (HNSCC) who experienced a near-complete histologic response after receiving 13 days of neoadjuvant erlotinib treatment [6]. Whole exome sequencing of the pre-treatment tumor revealed a *MAPK1*^E322K^ somatic mutation, which was further implicated in mediating erlotinib sensitivity by preclinical studies.

*MAPK1* encodes ERK2, which will be referred to as MAPK1, a component of the mitogen activated signaling (MAPK) pathway downstream of RAS, RAF and MEK. The *MAPK1*^E322K^ hotspot mutation, which causes constitutive activation of ERK2 [7, 8], occurs in approximate 1.3% of HNSCC [9, 10] and in 8% of cervical squamous cell carcinomas [11].

We reported that HNSCC cells harboring an endogenous *MAPK1*^E322K^ mutation (HSC-6) or HNSCC cells hemizygous for wild type *MAPK1* (FaDu) transfected with mutant *MAPK1*^E322K^ demonstrated enhanced EGFR phosphorylation and activation of downstream signaling [6, 7, 12]. FaDu cells expressing exogenous mutant *MAPK1* demonstrated increased senescence to erlotinib compared with those expressing exogenous wild type *MAPK1* or vector control [6]. These findings suggest potential crosstalk between mutant MAPK1 and EGFR signaling pathways. However, the molecular mechanism underlying this crosstalk remains unknown.

Previous studies demonstrated ERK activity results in the production of the EGFR ligand amphiregulin (AREG) in airway epithelial cells [13] [14]. More recently, MAPK1 specifically and not ERK1 was reported to be required for AREG production in HNSCC cells [15]. Increased AREG levels have been associated with enhanced response to EGFR TKIs in *EGFR* wild-type cancer cell lines and patient tumors [16, 17]. We previously reported that increased secretion of AREG in HNSCC is critical for EGFR crosstalk and transactivation [18]. The present study was undertaken to test the hypothesis that MAPK1^E322K^ increases sensitivity to erlotinib through enhanced AREG-EGFR activation in HNSCC.

## RESULTS

### MAPK1^E322K^ is associated with increased secretion of AREG in HNSCC cells

**Figure 1:**
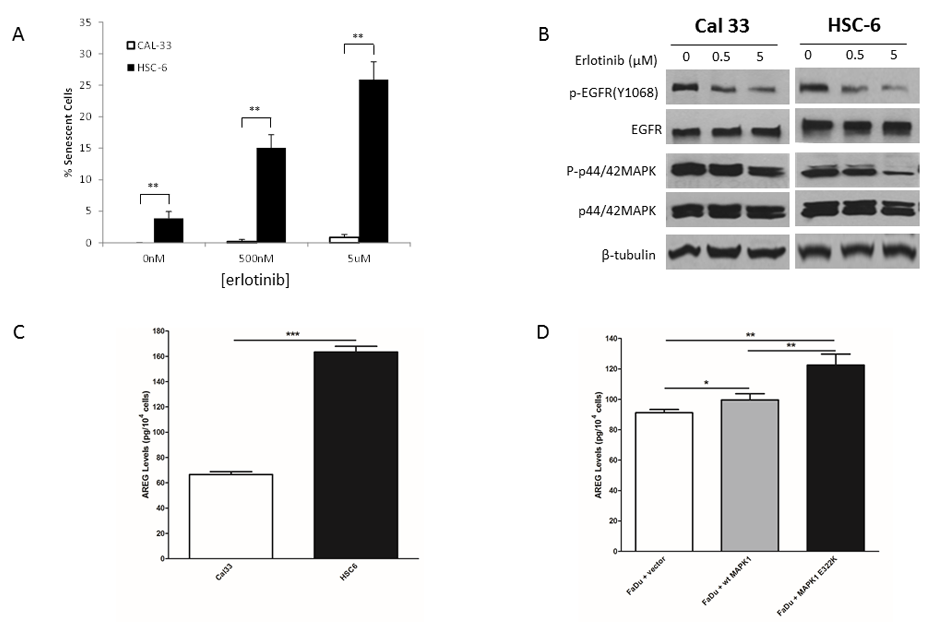
*MAPK1*^**E322K**^ was associated with increased secretion of AREG and enhanced response to erlotinib in HNSCC cells **A.** Following 48 h treatment with erlotinib (500 nM or 5 μM) or vehicle control, HSC-6 cells had enhanced erlotinib-induced senescence compared to Cal33 cells. **B.** Cal33 and HSC-6 cells were treated for 48 h treatment with erlotinib (500 nM or 5 μM) or vehicle control and cell lysates were analyzed by immunoblotting. P-EGFR levels were diminished in both cell lines following erlotinib treatment. p-MAPK levels demonstrated modest reduction in Cal-33 cells and pronounced reduction in HSC-6 cells following erlotinib treatment. **C.** *MAPK1*^E322K^ HSC-6 cells secrete higher levels of AREG compared to *MAPK1*^WT^ Cal33 cells. **D.** Expression of exogenous *MAPK1*^E322K^ increased AREG secretion compared with vector-control or wild type transfected *MAPK1* in FaDu (MAPK1-hemizygous wild type) engineered cells (*n* = 3). Similar results were obtained with triplicate wells in three independent experiments. *p < 0.05, **p < 0.01, ***p < 0.001.

We previously reported that HSC-6 cells harboring endogenous *MAPK1*^E322K^ expressed higher basal level of p-EGFR (Y1068) compared to wild-type *MAPK1*(*MAPK*1^WT^) Cal33. In addition, FaDu cells engineered to express exogenous *MAPK1*^E322K^ expressed high basal level of p-EGFR (Y1068) compared to FaDu cells exogenously expressing *MAPK1*^WT^ or vector control, suggesting that the activating MAPK1 mutation likely causes a feedback activation of EGFR in HNSCC cells. We determined that *MAPK1*^E322K^ HSC-6 cells were more sensitive to erlotinib than *MAPK1*^WT^ Cal33 cells as assessed by senescence assay (Figure 1A). Representative pictures of senescence staining are presented in Figure S1. Following treatment with erlotinib, P-EGFR and P-MAPK levels were reduced in both Cal33 and HSC-6 cells (Figure 1B), demonstrating inhibition of EGFR under experimental conditions. We previously reported that FaDu cells engineered to exogenously express *MAPK1*^E322K^ were more sensitive to erlotinib than FaDu cells engineered to express *MAPK1*^WT^ or vector control using this same senescence assay [6].

We hypothesized that MAPK1^E322K^ activated EGFR through enhanced EGFR ligand secretion. To test this hypothesis, we first measured the release of several EGFR autocrine ligands in HNSCC cells including AREG, TGF-α, EGF and HB-EGF (Table S1). We found that endogenous *MAPK1*^E322K^ HSC-6 cells secreted higher AREG levels compared with *MAPK1*^WT^ Cal33 cells (Figure 1C). To assess AREG secretion in the same genetic background we tested FaDu cells engineered to exogenously express *MAPK1*^E322K^, *MAPK1*^WT^ or vector-control and found that cells expressing *MAPK1*^E322K^ had increased AREG secretion (Figure 1D). These results suggest that MAPK1^E322K^ may be involved in an autocrine loop of AREG production leading to increased p-EGFR *via* ligand-dependent activation. Expression of exogenous *MAPK1*^WT^ also appeared to modestly increase AREG secretion, suggesting that overexpression of wild type *MAPK1* may also participate in this process, albeit to a lesser degree than *MAPK1*^E322K^.

To determine if the effects of MAPK1^E322K^ were specific to AREG, we also examined secretion of other autocrine EGFR ligands in the conditioned media of Cal33, HSC-6 and FaDu engineered cells. Unlike AREG, MAPK1^E322K^ did not induce detectable secretion of TGF-α, EGF or HB-EGF, which were below the limits of assay detection for all samples (Table S1), thus excluding the possible involvement of these other EGFR ligands.

### MAPK1 inhibition decreases AREG secretion

**Figure 2:**
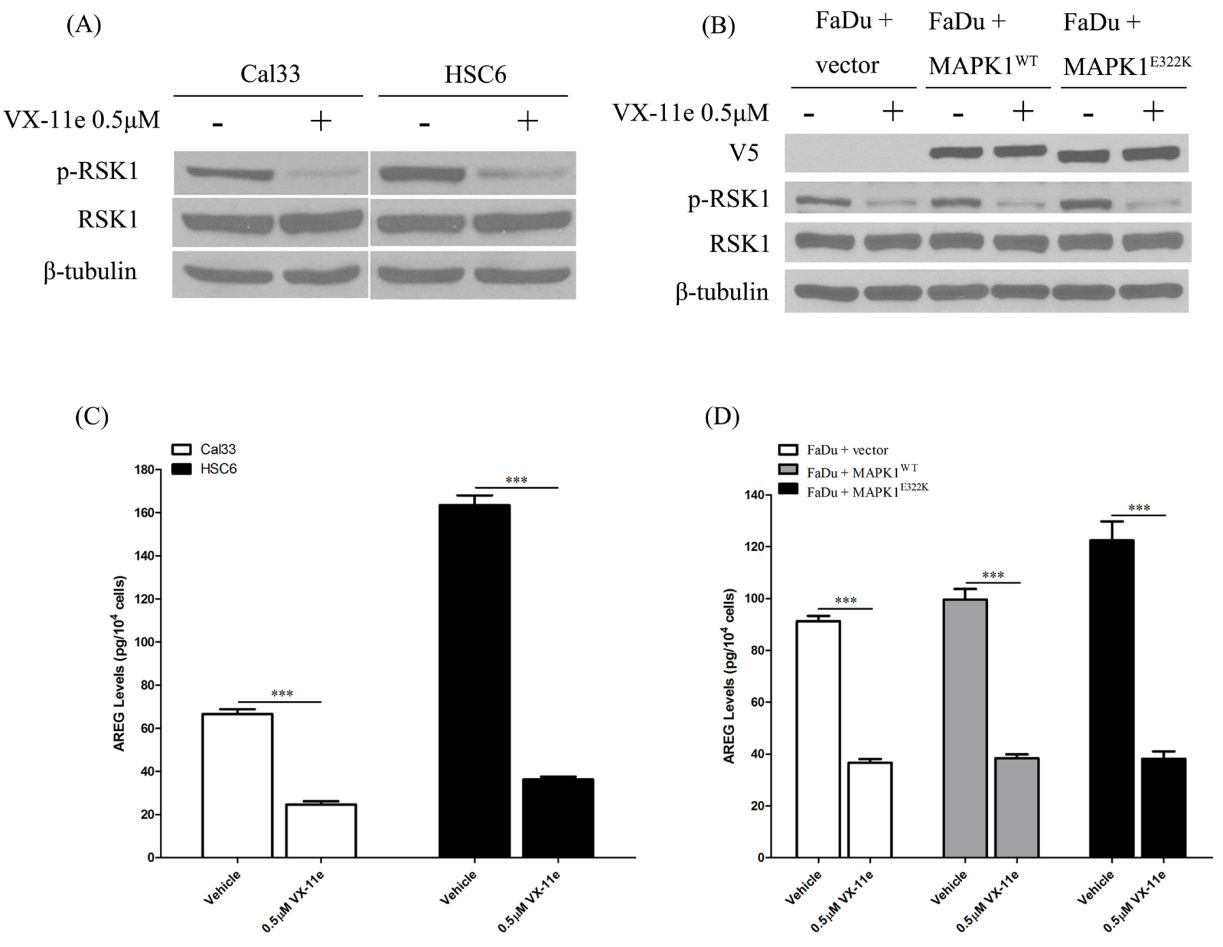
MAPK1 inhibition decreased AREG secretion in HNSCC, especially in the cells harboring the *MAPK1*^**E322K**^ mutation **A.** HNSCC cells were treated with DMSO vehicle or VX-11e (0.5 μM) for 24 h and cell protein extracts analyzed. VX-11e inhibited phosphorylation of RSK1 in Cal33 and HSC-6. **B.** VX-11e inhibited phosphorylation of RSK1 in FaDu engineered cells. **C.** For quantification of secreted AREG, cells were pretreated with DMSO vehicle or VX-11e (0.5 μM) for 2 hours, washed with 1× PBS, then treated for 24 h. Conditioned media was collected and assayed by ELISA. VX-11e decreased AREG secretion in Cal33 and HSC-6 cells as well as FaDu engineered cells **D.**. The extent of AREG decrease in endogenous *MAPK1*^E322K^ HSC-6 and FaDu cells engineered to express *MAPK1*^E322K^ was higher than respective endogenous *MAPK1*^WT^ Cal33 and FaDu cells with vector or wild type *MAPK1* transfection. (*n* = 3. *p < 0.05, **p < 0.01). Similar results were obtained with triplicate wells in three independent experiments.

To determine the role of MAPK1^E322K^ in AREG secretion, HNSCC cells were treated with a dual MAPK1 (ERK2) and ERK1 kinase inhibitor, VX-11e [19]. Ribosomal protein S6 kinase (RSK1) is a MAPK1 (ERK2)-specific downstream substrate [19, 20]. As a pharmacodynamic measure of VX-11e inhibition, we evaluated the effects on RSK1 phosphorylation. As shown in Figures 2A and 2B, VX-11e inhibited phosphorylation of RSK1 in HNSCC cells. To test our hypothesis that MAPK1 inhibition would decrease AREG secretion, we treated cells with VX-11e (0.5 μM) or DMSO vehicle control and collected the conditioned media. We found that AREG levels were substantially decreased following treatment with VX-11e in Cal33, HSC6 and FaDu engineered cells (Figures 2C and 2D). HNSCC cells harboring the *MAPK1*^E322K^ mutation had the largest decrease in AREG levels following VX-11e treatment. These results indicate that AREG production in HNSCC cells is dependent upon MAPK signaling and that the MAPK1^E322K^-induced AREG autocrine loop was significantly attenuated by MAPK1 inhibition. These findings implicate MAPK1^E322K^ in the enhanced production of AREG in HNSCC.

**Figure 3:**
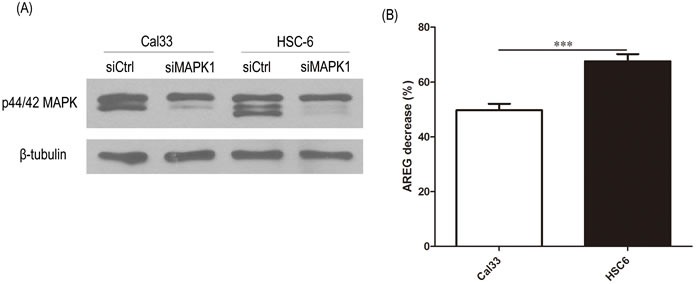
siRNA knockdown MAPK1 significantly decreased secretion of AREG in HSC-6 cells expressing mutant *MAPK1*^**E322K**^ compared with Cal33 cells expressing *MAPK1*^**WT**^ 48 hours after transfection with control or *MAPK1*-targeting siRNA, CAL33 and HSC-6 cells were trypsinized and seeded into 48-well plates. 24 hours later supernatants were analyzed by ELISA and protein isolates analyzed. **A.** *MAPK1* siRNA reduced total MAPK1 expression levels compared with non-targeting control siRNA. **B.** MAPK1 knockdown by siRNA reduced secretion of AREG to a greater extent in *MAPK1*^E322K^ HSC-6 cells than in *MAPK1*^WT^ Cal33 cells. Similar results were obtained with triplicate wells in three independent experiments.

In addition to pharmacologic inhibition of MAPK with VX-11e, we also examined AREG secretion levels in endogenous *MAPK1*^E322K^ HSC-6 cells and endogenous *MAPK1*^WT^ Cal33 cells upon MAPK1 knockdown by siRNA. As shown in Figure 3, *MAPK1* siRNA efficiently reduced total MAPK1 (ERK2) expression levels and led to a reduced secretion of AREG compared to the non-targeting siRNA control. The decrease in AREG production following knockdown was greater in *MAPK1*^E322K^ HSC-6 cells than *MAPK1*^WT^ Cal33 cells.

### MAPK1 inhibition decreases sensitivity to erlotinib in *MAPK1*^E322K^ mutant HNSCC cells, while exogenous AREG restores erlotinib sensitivity

**Figure 4:**
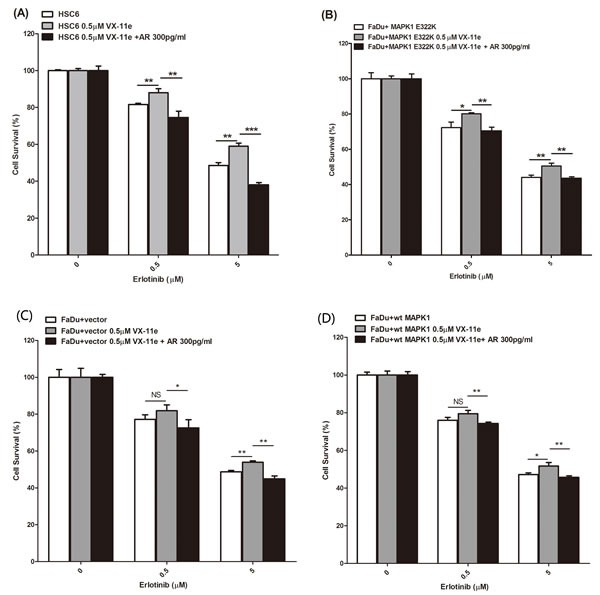
MAPK1 inhibition decreased sensitivity to erlotinib in *MAPK1*^**E322K**^ mutant HNSCC cells, while exogenous AREG restored erlotinib sensitivity HSC-6 cells and FaDu cells engineered to express *MAPK1*^E322K^ were pretreated with DMSO, VX-11e (0.5 μM) or combination of VX-11e (0.5 μM) and AREG (300 pg/ml) for 24 hours. Cells were then treated with two concentrations of erlotinib or a combination of VX-11e (0.5μM) and erlotinib or the triple combination of VX-11e (0.5 μM) and AREG (300pg/ml) and erlotinib for 48 hours. Cell survival was measured by crystal violet dye extraction growth assay and plotted relative to DMSO vehicle control (erlotinib alone) or VX-11e control (combination of VX-11e and erlotinib) or VX-11e and AREG control (combination of VX-11e, AREG and erlotinib). **A.** MAPK1 inhibition by VX-11e decreases sensitivity to erlotinib in HSC-6 cells, while exogenous AREG restored sensitivity. **B.** MAPK1 inhibition by VX-11e decreased sensitivity to erlotinib in FaDu cells engineered to express *MAPK1*^E322K^, while exogenous AREG restored its sensitivity. Decreased sensitivity to erlotinib with MAPK1 inhibition was attenuated in vector control transfected FaDu cells **C**. and FaDu cells engineered to express *MAPK1*^WT^ **D**. (*n* = 3. *p < 0.05, **p < 0.01, ***p < 0.001). Similar results were obtained with triplicate wells in three independent experiments.

We next evaluated whether inhibition of MAPK signaling by VX-11e would decrease sensitivity to erlotinib in the HSC-6 and FaDu engineered cells expressing exogenous *MAPK1*^E322K^. We first pre-treated the cells with VX-11e for 24 hours to inhibit MAPK1^E322K^ -driven autocrine AREG loop, then treated the cells with vehicle, VX-11e, erlotinib or a combination of VX-11e and erlotinib (0.5 or 5μM). As shown in Figures 4A and 4B, inhibition of ERK1/2 by VX-11e decreased sensitivity to erlotinib in HSC-6 and FaDu engineered cells expressing exogenous MAPK1^E322K^. Given that MAPK1^E322K^ was associated with increased AREG secretion and, conversely, inhibition of MAPK1 results in reduced AREG secretion and decreased sensitivity to erlotinib, we next evaluated ability of exogenous AREG to restore erlotinib sensitivity in HSC-6 cells and FaDu engineered cells expressing exogenous *MAPK1*^E322K^ under conditions of MAPK1 inhibition. We determined that AREG supplementation at 300 pg/ml, which was within the range of observed secreted AREG levels (Table S1), approximately restored AREG levels to baseline under these conditions. To test the effects of exogenous AREG on MAPK1 inhibition in HSC-6 cells and FaDu engineered cells expressing exogenous *MAPK1*^E322K^, we pretreated cells with a combination of AREG and VX-11e for 24 hours, then changed media and treated with combinations of erlotinib (0, 0.5 or 5μM), AREG and VX-11e for 48 hours. We observed that the sensitivity to erlotinib was restored by exogenous AREG in those cells under conditions of MAPK1 inhibition (Figure 4A and 4B). These same studies employing FaDu engineered control cells or FaDu cells expressing wild type *MAPK1*demonstrated attenuated resistance to erlotinib in the presence of VX-11e (Figure 4C and 4D) compared to *MAPK1*^E322K^ expressing cells. Collectively, MAPK1^E322K^ drove increased secretion of AREG, which created an enhanced autocrine feedback loop involving AREG, EGFR, and ERK signaling compared with wild type MAPK1. Sensitivity to erlotinib is likely a matter of degree of dependency upon this autocrine loop.

### Knockdown of AREG decreases EGFR-MAPK pathway activation

**Figure 5:**
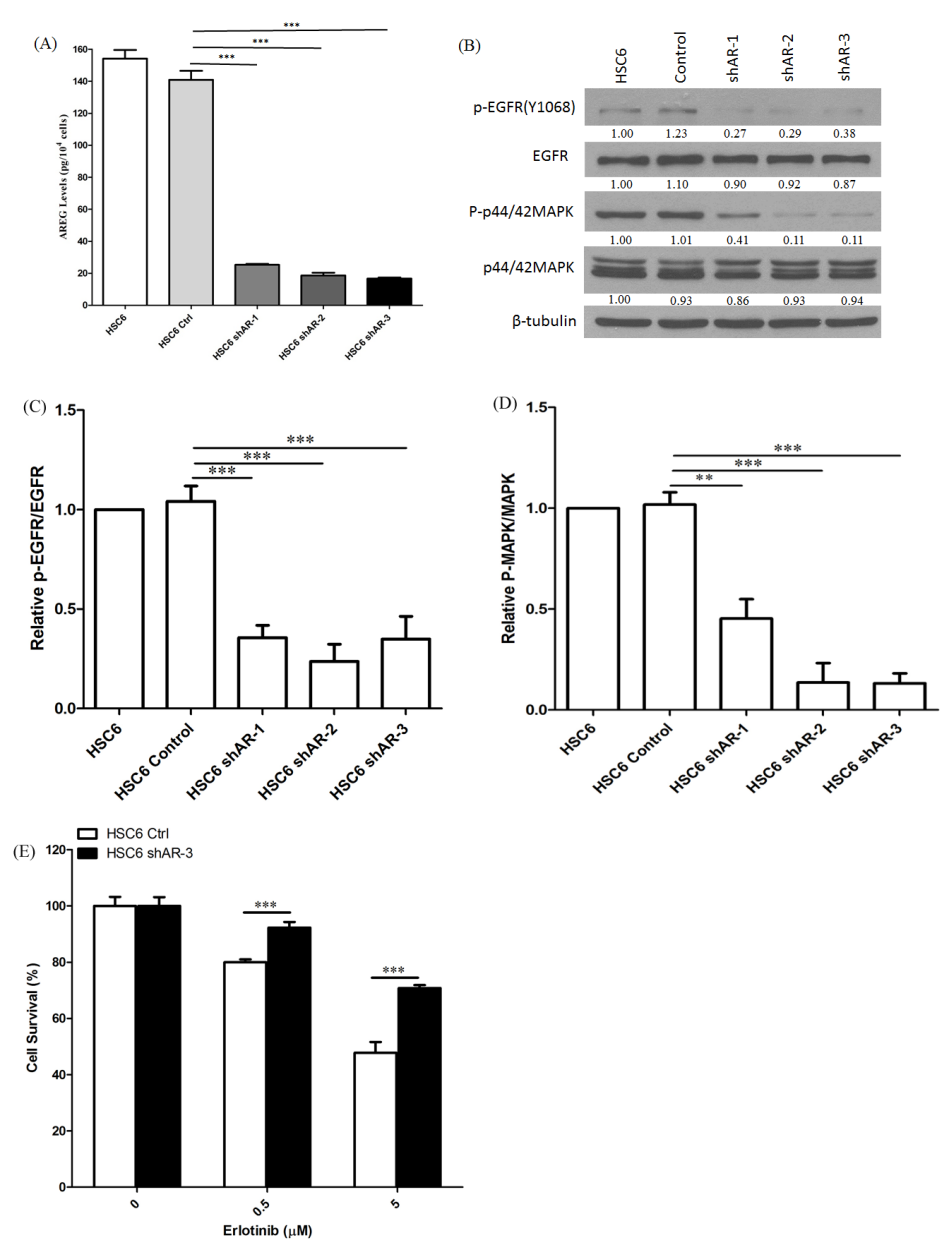
Knockdown of AREG reduced AREG secretion and EGFR-MAPK pathway activation **A.** Conditioned media from HSC-6 cells transfected with GFP control or shAREG was analyzed by ELISA. Assays were performed in triplicate. ***p < 0.001. Similar results were obtained with triplicate wells in three independent experiments. **B.** AREG knockdown lead to decreased expression of P-EGFR (Y1068) and P-p42/44 MAPK in HSC-6 cells by immunoblotting. **C.** Densitometry analysis of EGFR phosphorylation. P-EGFR was normalized to EGFR as a loading control. Cumulative results are shown from three independent experiments. ***p < 0.001. **D.** Densitometry analysis of MAPK phosphorylation. P-p42/44 MAPK was normalized to p42/44 MAPK. Cumulative results from three independent experiments are shown. **p < 0.01, ***p < 0.001. **E.**. Depletion of AREG by shRNA decreased erlotinib sensitivity in *MAPK1*^E322K^ cells. HSC-6 cells expressing GFP-control or shAREG were treated with erlotinib at the indicated concentrations. Cell survival was measured by crystal violet dye extraction growth assay and plotted relative to DMSO vehicle control. (*n* = 3, ***p < 0.001). Similar results were obtained in three independent experiments as well as other HSC-6 cell clones with shAREG knockdown.

To further test the contribution of AREG production to erlotinib sensitivity in the setting of *MAPK1*^E322K^, we used shRNA to knockdown AREG expression. We first generated HSC-6 cells with stable green fluorescent protein (GFP) control or AREG shRNA expression by lentiviral particle transduction. As shown in Figure 5A, HSC-6 cells with AREG knockdown secreted much lower levels of AREG compare to controls. AREG knockdown in HSC-6 cells also reduced expression levels of p-EGFR (Y1068) and p-p44/42 MAPK (Figures 5B-5D). Therefore, EGFR signaling activation in cells harboring *MAPK1*^E322K^ is dependent on AREG secretion.

### AREG knockdown decreases erlotinib sensitivity in *MAPK1*^E322K^ cells

To determine the contribution of AREG production to erlotinib sensitivity in *MAPK1*^E322K^ mutated HNSCC models, we compared erlotinib responses in HSC-6 cells in the setting of AREG knockdown by shRNA compared with GFP-control. As shown in Figure 5E, HSC-6 cells with shAREG knockdown were significantly less sensitive to erlotinib compared to GFP-control. These data strongly indicate that MAPK1-driven AREG production mediates activation of cellular EGFR signaling and confers sensitivity to erlotinib in the setting of *MAPK1*^E322K^.

Given that MAPK1^E322K^ increased AREG secretion and knockdown of AREG resulted in decreased erlotinib sensitivity, we next evaluated the ability of exogenous AREG to restore erlotinib sensitivity in HSC-6 cells under conditions of AREG knockdown. To test the effects of exogenous AREG under conditions of AREG knockdown, we pretreated cells with AREG for 24 hours, then treated cells with combinations of erlotinib (0, 0.5 or 5μM) and AREG for 48 hours. We compared cell viability in the presence or absence of supplemental AREG, and we observed that sensitivity to erlotinib was enhanced by exogenous AREG in HSC-6 cells under conditions of AREG knockdown (Figure S2).

### Depletion of AREG expression in *MAPK1*^E322K^ cells reduces tumor growth and sensitivity to erlotinib *in vivo*

**Figure 6:**
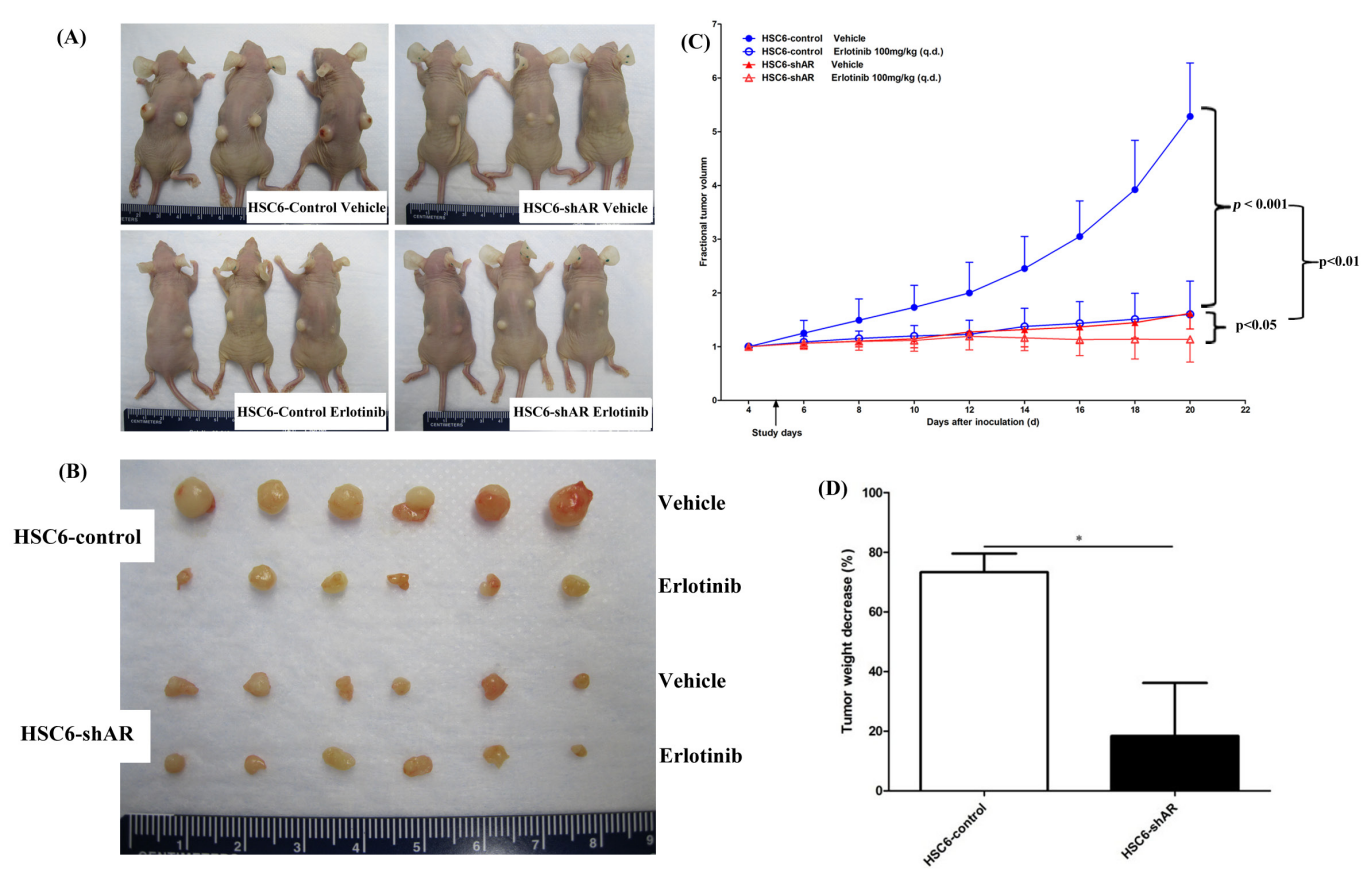
Depletion of AREG by shRNA decreased erlotinib sensitivity in *MAPK1*^**E322K**^ xenografts HSC-6 cells expressing GFP-control or AREG shRNA were selected and expanded. 2×10^6^ cells were inoculated subcutaneously into both flanks of 5-6 week-old female nude mice (*n* = 6 per group). Four days after inoculation, tumor volumes were measured and the mice were divided into vehicle or erlotinib treatment groups. Erlotinib was administered at a dosage of 100 mg/kg daily by oral gavage. 10% HPBCD was administrated as vehicle control. Tumor volumes were measured every other day. **A.** Tumor bearing mice and **B.** excised tumors are shown. **C.** Erlotinib significantly suppressed tumor growth in HSC-6-control xenografts. (***p < 0.001). The erlotinib effect was significant but more modest in HSC-6-shAR xenografts (*p < 0.05). **D.** Tumor weights were significantly decreased by erlotinib in HSC-6-control xenografts to a greater degree than HSC-6-shAR xenografts (*p < 0.05).

Expression of *MAPK1*^E322K^ was associated with increased secretion of AREG, and knockdown of AREG in *MAPK1*^E322K^ cells resulted in an expected decrease in AREG secretion with concomitant diminished sensitivity to erlotinib *in vitro*. To validate these findings *in vivo*, we assessed the antitumor efficacy of erlotinib in endogenous *MAPK1*^E322K^ HSC-6 mouse xenografts (control) and shAREG knockdown HSC-6 mouse xenografts. Consistent with our *in vitro* results, tumor growth was significantly suppressed in HSC-6 xenografts without AREG depletion (HSC-6-control groups) with erlotinib treatment (100 mg/kg) compared with vehicle control (*p* < 0.001) (Figure 6). Knockdown of AREG alone was associated with a suppression of tumor growth that was similar to that observed with erlotinib treatment of HSC-6 control xenografts (Figure 6C). The erlotinib effect was modest though significant in AREG depleted tumors (*p* < 0.05, Figure 6C). As shown in Figure 6C, the anti-tumor effects of erlotinib were significantly greater for HSC-6-control xenografts than HSC-6-shAR xenografts (*p* < 0.01), indicating that depletion of AREG decreased response to erlotinib in the setting of *MAPK1*^E322K^. Similarly, a more marked tumor weight reduction was observed upon erlotinib treatment in the HSC-6-control group *versus* the HSC-6-shAR group (*p* < 0.05, Figure 6D).

## DISCUSSION

EGFR TKIs are effective in non-small cell lung cancer (NSCLC) patients with an activating mutation of the EGFR TK domain [21-23]. These mutations are not found in HNSCC and to date, predictive biomarkers for TKIs have been lacking in this malignancy. We recently reported a case of an HNSCC tumor harboring a *MAPK1*^E322K^ somatic mutation that contributed to an exceptional clinical response to single agent erlotinib [6]. MAPK1 activity has generally been reported to be associated with resistance to EGFR TKIs in cancers other than HNSCC. *MAPK1* amplification, which may also activate ERK signaling, leads to increased EGFR internalization through Thr-669, and confers EGFR TKI resistance in *EGFR* mutant NSCLC [24]. Similarly, in preclinical models of pancreatic cancer and lung cancer, inhibition of *MAPK1* expression by siRNA or MAPK1 activity by MEK inhibitors sensitized specific cancer cell lines to erlotinib [25, 26]. Thus, the effect of two different ERK genomic alterations (point mutation or amplification of *MAPK1*) may have distinct clinical implications in different types of cancer. The paradoxical presence of an erlotinib sensitivity-conferring MAPK1 activating mutation in HNSCC warranted further mechanistic studies of this mutation.

We previously established that HNSCC cells harboring *MAPK1*^E322K^ showed increased activation of EGFR and downstream signaling compared to *MAPK1*^WT^ cells[6]. In the present study, we focused on further elucidating the mechanism underlying this exceptional erlotinib response. Our results indicate that endogenous *MAPK1*^E322K^ was associated with higher secreted AREG levels compared to endogenous *MAPK1*^WT^. Exogenous expression of *MAPK1*^E322K^ also increased AREG secretion compared to vector control or *MAPK1*^WT^ expression in an engineered HNSCC model. Consistent with literature findings that enhanced secretion of AREG leads to hyperactivation of EGFR signaling [27, 28], our results support the hypothesis that MAPK1^E322K^ drives increased secretion of AREG, creating an enhanced feedback autocrine loop involving AREG, EGFR and downstream signaling. This conclusion is further supported by evidence that MAPK1 targeting resulted in reduced secretion of AREG in the *MAPK1*^E322K^ cell line.

AREG, a ligand of EGFR, is synthesized as a transmembrane precursor that undergoes a series of proteolytic processes to yield a mature secreted form [27]. The binding of AREG to EGFR induces autophosphorylation of the EGFR intracellular tyrosine kinase domain, which activates the downstream signaling pathways, including the MAPK pathway. Consequently, growth, proliferation, migration, or invasiveness are enhanced after the activation of EGFR [29].

Our results demonstrated that MAPK1^E322K^ increased AREG secretion. This dependency on AREG-EGFR signaling conferred erlotinib sensitivity, while depletion of AREG reduced EGFR signaling and lead to decreased sensitivity to erlotinib *in vitro* and *in vivo*. It has been reported that autocrine ligand production can predict sensitivity to gefitinib in wild type EGFR cancers (10 NSCLC lines and 4 HNSCC lines), as gefitinib was significantly more effective at inhibiting the growth of high AREG-producing cell lines compared with the low AREG-producing cells[16]. Rogers *et al*. also reported that AREG secretion showed a positive correlation with sensitivity to gefitinib in a panel of 18 HNSCC cell lines [30]. A study comparing AREG expression in 24 *EGFR* wild type NSCLC patients found AREG expression was significantly higher in NSCLC patients who developed stable disease following gefitinib or erlotinib treatment compared with those who developed disease progression [16]. Another study in 73 WT *EGFR* NSCLC showed that overall survival and progression-free survival were significantly longer in AREG-positive patients compared to AREG-negative patients[17]. Exploratory molecular analyses of a phase II trial in pancreatic carcinoma, showed patients with high baseline serum AREG levels might benefit from erlotinib [31].

In contrast, increased levels of serum AREG have been correlated with a lack of benefit from gefitinib treatment in patients with advanced NSCLC [32, 33], and in an independent study AREG overexpression was reported to promote resistance to gefitinib-induced apoptosis rather than sensitivity in *KRAS* mutant NSCLC cell lines [34, 35]. These discrepancies may be a result of different cancer types, use of different cell lines, heterogeneous methods used to detect AREG expression, and/or differences in AREG concentrations in the local tumor microenvironment and the systemic circulation. Our results are consistent with increased AREG secretion leading to *EGFR* signaling-dependency and erlotinib sensitivity in wild type EGFR HNSCC.

There are limitations to this study. To our knowledge only one cell line with endogenous *MAPK1*^E322K^ has been identified to date according to the literature, the Cancer Cell Line Encyclopedia and the Catalogue of Somatic Mutations in Cancer [12, 36]. The study of additional cell lines harboring this same mutation would be more powerful. The use of engineered cell lines provide the opportunity to evaluate the effects of the mutation given a specific context. However, these cells lack a natural selection history that likely accompanies an advantage-conferring mutation. Therefore, results may not exactly phenocopy cell lines harboring endogenous *MAPK1*^E322K^. Given these caveats, the mechanism of enhanced AREG secretion and increased erlotinib sensitivity that accompanies *MAPK1*^E322K^ in the engineered and endogenous cell line studies is compelling. It will be important to further elucidate the mechanisms contributing to MAPK1^E322K^ and AREG enhanced erlotinib sensitivity in order to molecularly define patients who may be candidates for EGFR TKI treatment in addition to those whose tumors harbor this mutation.

In summary, our studies revealed an essential role for MAPK1^E322K^ in mediating erlotinib sensitivity. We identified autocrine AREG-EGFR signaling as mediating the erlotinib sensitivity resulting from MAPK1^E322K^. Thus, activation of AREG-EGFR signaling might confer erlotinib treatment benefit for the subset of patients with tumors harboring *MAPK1*^E322K^ mutation.

## MATERIALS AND METHODS

### Cell culture

Three HNSCC cell lines were selected based on their *MAPK1* genotype: FaDu (*MAPK1* hemizygous wild type), Cal33 (*MAPK1* wild type (*MAPK1*^WT^)) and HSC-6 (*MAPK1*^E322K^). The HSC-6 cell line was a kind gift from Prof. Johji Inazawa (Tokyo Medical and Dental University, Japan). A set of engineered FaDu cell lines exogenously expressing GFP, *MAPK1*^WT^ or *MAPK1*^E322K^ were generated by retrovirus transduction. Cells were maintained in Dulbecco's modified Eagle's medium (Mediatech, Manassas, VA, USA) supplemented with 10% inactivated fetal bovine serum (FBS; Lonza, Walkersville, MD, USA) and 1% penicillin/streptomycin (Mediatech). Cells were incubated at 37°C with 5% CO_2_.

### Reagents and antibodies

Erlotinib (Tarceva™) was obtained from OSI Pharmaceuticals (Uniondale, NY). The MAPK1 inhibitor VX-11e was purchased from MedChem Express, and was purchased from Peprotech. Crystal Violet was purchased from Sigma. The following antibodies were purchased from commercial sources: anti-phospho-EGFR (Tyr1068; CST-3777s); anti-EGFR (BD Biosciences-610017); anti-p44/42 MAPK (CST-9102s); anti-phospho-p44/42 MAPK (CST-4370s); anti-phospho-Rsk1 (EMD Millipore-04-419); anti-RSK (BD Biosciences-610226); anti-V5 tag (Abcam-ab27671) and anti-beta-Tubulin (Abcam-ab6046).

### Senescence assay

β-galatosidase activity at pH 6 was detected in senescent cells by light microscopy (100x) following staining using the senescence staining kit (Cell Signaling Technology, USA). Number of senescent cells and total number of cells per field were analyzed for at least 5 fields for each cell type and treatment conditions. Representative results from 3 independent experiments are presented. Representative staining pictures are provided in Figure S1.

### Western blotting analysis

Western blot analyses were performed as previously described [37].

### ELISAs

The concentration of secreted AREG protein in cultured media was determined using the AREG Quantikine ELISA kit (R&D Systems-DAR00). The concentrations of TGF-α (R&D Systems-DTGA00), EGF (R&D Systems-DEG00) and HB-EGF (Abcam-ab100531) were also measured in the same conditioned media using specific Quantikine ELISA kits. In brief, 2×10^4^ cells/well were seeded in the 48-well plates and cultured overnight. Cells were then washed with 1×phosphate-buffered saline (PBS) once and incubated in 0.5 ml of complete medium for 24 h. The media were collected and centrifuged and supernatant was collected and stored at −80°C prior to analysis. Cells were trypsinized and counted. ELISA assays were performed according to the manufacturer's protocols. Samples were run in triplicate, and secreted AREG was normalized against standard curves and presented as pg per 1×10^4^ cells.

### Viral transduction

To generate retroviruses, plasmids encoding GFP (vector control), MAPK1^WT^ or MAPK1^E322K^ were transfected into Platinum-A cells using FuGENE HD Transfection Reagent (Promega, Madison, USA). Virus particles were collected 72 hours post transfection. For viral infection, targeted cells were infected on three consecutive dates with virus in fresh complete medium containing Polybrene (14 μg/ml; Sigma, St Louis, MO, USA) then replaced with fresh medium. To generate AREG knockdown in cells, HSC-6 cells were infected with AREG-targeting short-hairpin RNA (shRNA) lentiviral particles (sc-39412-V, Santa Cruz Biotechnology, USA) and selected by puromycin according to the manufacturer's protocols. Cell colonies expressing AREG shRNA were selected and assayed by enzyme-linked immunosorbant assays (ELISA) and Western blotting.

### siRNA transfection

Cal33 and HSC-6 cells were transfected with 5 uM non-targeting small interfering RNA (siRNA) (OriGene-SR30004) or MAPK1-targeting siRNA (OriGene-SR303751) using the siTran1.0 siRNA Transfection Reagent (OriGene-TT300001) according to the manufacturer's protocol. Briefly, cells were apportioned into T25 flasks and transfected 24 hours later with 5 uM control siRNA or *MAPK1*-targeting siRNA. At 48 hours after transfection, cells were trypsinized and seeded into 48-well plates. ELISA quantification was performed using 24-hour conditioned media. In parallel, cells protein samples were isolated, and the efficiency of knockdown was analyzed by western blotting.

### Cell proliferation assay

The effects of erlotinib on cell line proliferation were determined by crystal violet dye extraction growth assay. Briefly, exponentially growing cells were seeded into 12-well plates at a low cell density of 2.5×10^4^ cells/well in complete medium, cultured overnight and treated with indicated drugs. Crystal violet dye extraction growth assays were performed at the end of treatment. Cells were washed twice with 1× PBS, fixed with 96% ethanol solution for ten minutes at room temperature then stained with 0.1% crystal violet solution for 30 min at room temperature with gentle shaking. Stained cells were washed with distilled water 3 times. Crystal violet dye was extracted using 1% SDS and absorbance was determined at 570nm. Assays were performed in triplicate.

### *In vivo* studies

Animal studies were conducted following a protocol approved by institutional animal care and use committee of the University of Pittsburgh. HSC-6 cells expressing GFP or AREG shRNA were selected and expanded *in vitro*. Then, 2 million cells were inoculated subcutaneously into each flank of 5-6 week-old female nude mice (Harlan Laboratories). Beginning at four days following inoculation, tumor volumes were measured daily by dial caliper. Tumor volumes in mm^3^ were calculated using the formula: tumor volume = (length×width^2^)/2. Tumor-bearing mice inoculated with the same cancer cells were randomized into the 2 treatment groups (3 mice, 6 tumors per group) to receive vehicle or erlotinib. Erlotinib (final concentration 10 mg/ml) was dissolved in a solvent of 10% (2-Hydroxypropyl)-β-cyclodextrin (HPBCD, Sigma-H107) in sterile water. Erlotinib was administered at a dose of 100 mg/kg by oral gavage once daily. 10% of HPBCD was administrated as vehicle control. Mice were sacrificed when the study was terminated and tumor weights were measured.

### Statistical analysis

Student's *t*-test was used to analyze data from ELISA, cell proliferation, immunoblotting densitometry and *in vivo* xenograft experiments in GraphPad Prism software package (GraphPad Prism software, San Diego, CA, USA). Data were considered significant at *p* < 0.05. Representative data are presented as means ± SD.

## SUPPLEMENTARY FIGURES AND TABLE


