# Supplementary material for: MAPK1E322K mutation increases head and neck squamous cell carcinoma sensitivity to erlotinib through enhanced secretion of amphiregulin
Source: Oncotarget. 2016 Mar 18;7(17):23300–11. doi: 10.18632/oncotarget.8188 (PMC5029627; doi:10.18632/oncotarget.8188)
Supplement: Supplementary file 1 [file oncotarget-07-23300-s001.pdf]

MAPK1^{E322K} mutation increases head and neck squamous cell carcinoma sensitivity to erlotinib through enhanced secretion of amphiregulin

Supplemental Materials

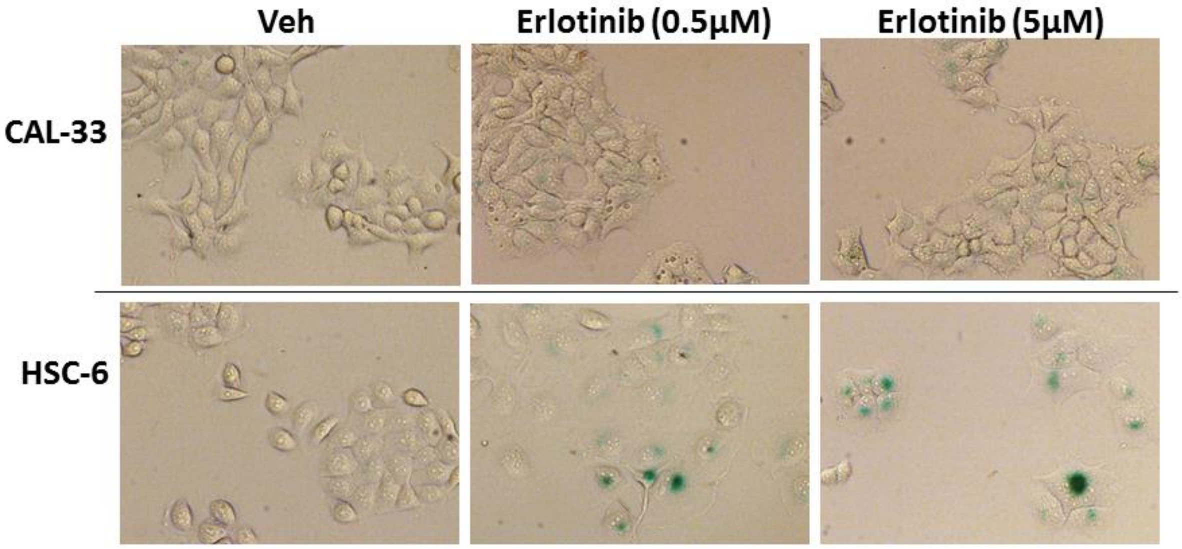

Figure S1. Representative pictures of enhanced senescence in HSC-6 cells compared with CAL33 following erlotinib treatment. Senescence staining of CAL-33 (*MAPK1* WT) and HSC-6 (*MAPK1* E322K) was performed following 48 h erlotinib treatment. β-galatosidase activity at pH 6 was detected in senescent cells by light microscopy (100x) following staining using the senescence staining kit (Cell Signaling Technology, USA).

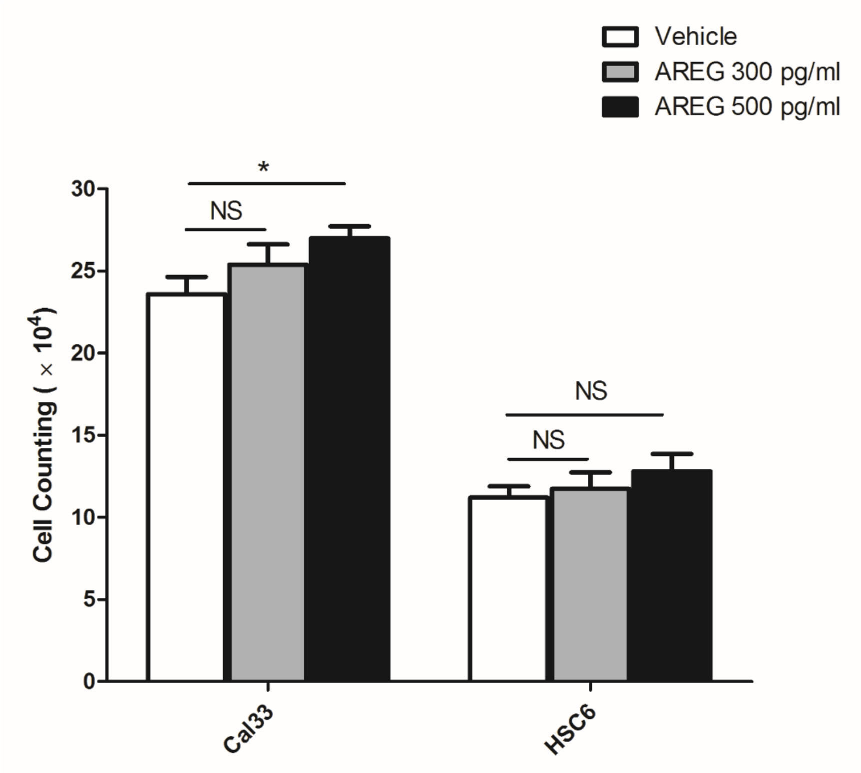

Figure S2. AREG at 300 pg/ml did not significantly increase cell growth of HNSCC cell lines. The effect of AREG supplementation on HNSCC cell viability were determined by trypan blue exclusion assay. Briefly, exponentially growing cells were seeded into 12-well plates at a cell density of 2.5×10^4 cells/well in complete medium, cultured overnight and treated with DMSO vehicle or AREG at indicated concentrations for 48 hours. NS: no significance; * $p<0.05$

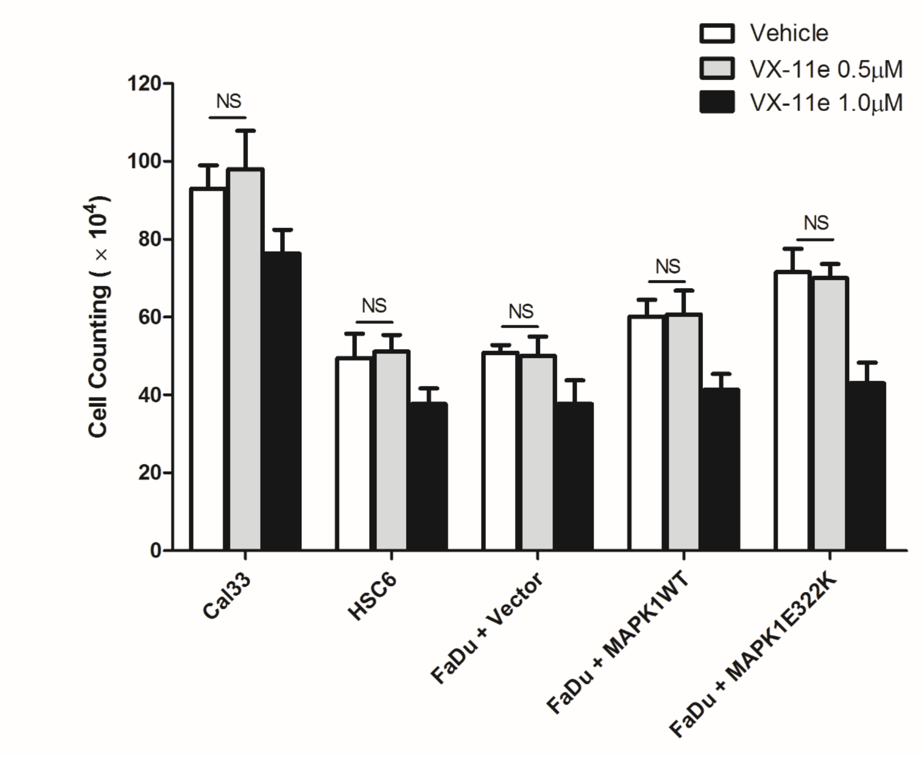

Figure S3. Dose response of VX-11e indicates 0.5μM dose did not reduce cell viability. The effects of VX-11e on HNSCC cell lines viability were determined by trypan blue exclusion assay. Briefly, exponentially growing cells were seeded into 6-well plates at a cell density of 2.5×10^5 cells/well in complete medium, cultured overnight and treated with DMSO vehicle or VX-11e at indicated concentrations for 24 hours. NS: no significance.

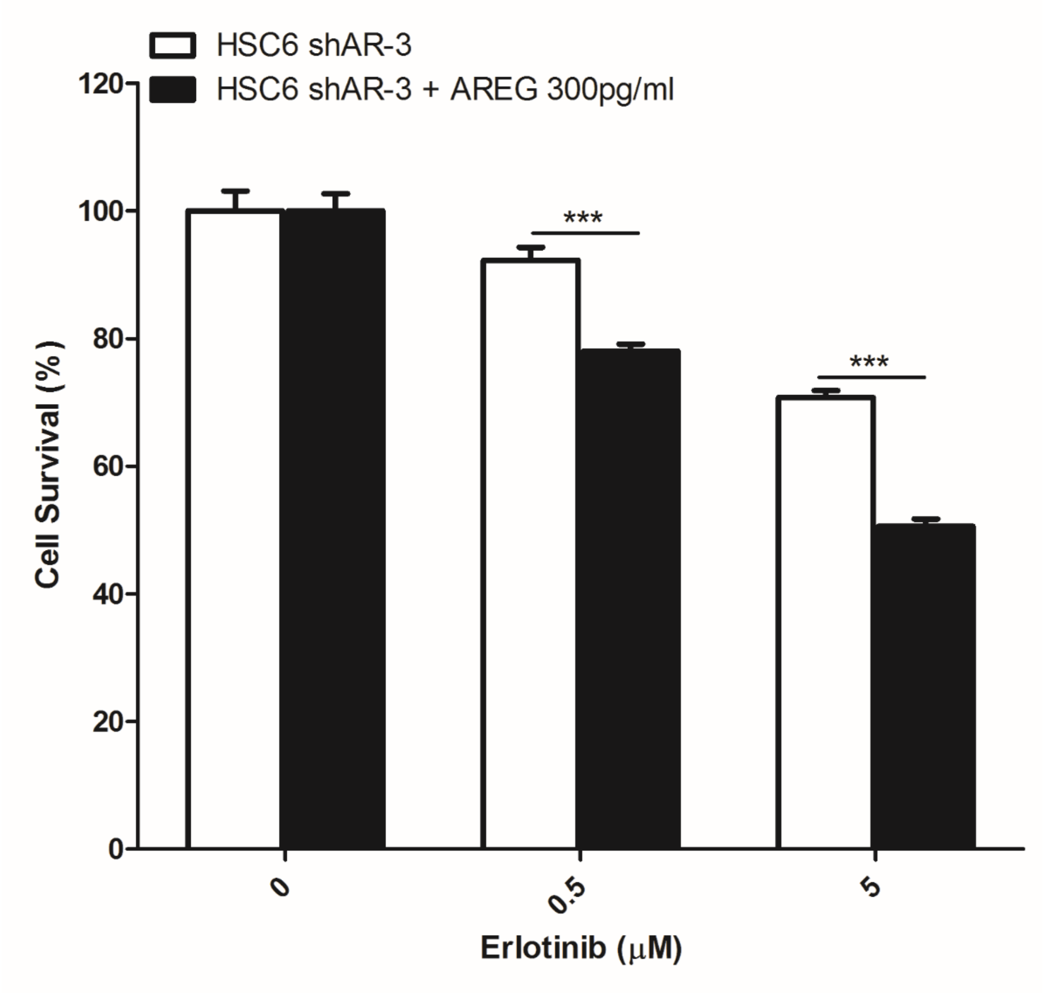

Figure S4. Exogenous AREG restores erlotinib sensitivity in *MAPK1*^{E322K} cells with depletion of AREG by shRNA. HSC-6 cells expressing shAR-3 were treated with erlotinib at the indicated concentrations for 48 hours. Cell survivals was measured by crystal violet dye extraction growth assay and plotted relative to DMSO vehicle control. (n=3, ***p<0.001). For exogenous AREG experiment, HSC-6 cells with shAR-3 knockdown were pretreated with AREG for 24 hours, then treated with combination of erlotinib (0, 0.5 or 5μmol/l) and AREG for 48 hours. Cell survival was measured by crystal violet dye extraction growth assay and plotted relative to AREG control (n=3, ***p<0.001). Similar results were obtained in three independent experiments as well as other HSC-6 cell clones with shAREG knockdown.

Table S1. EGFR Ligand levels in conditioned media from Cal33, HSC6 and FaDu engineered cells by quantitative ELISA

EGFR Ligands(pg/ml)	Cal33	HSC6	FaDu+ vector	FaDu+ *MAPK1wt*	FaDu+ $MAPK1^{E322K}$
AREG	399.7±13.4	568.8±15.6	282.8±6.4	298.8±12.3	343.0±20.3
TGF-α	<7.1	<7.1	<7.1	<7.1	<7.1
EGF	<3.9	<3.9	<3.9	<3.9	<3.9
HB-EGF	<20	<20	<20	<20	<20

Threshold sensitivities for AREG, TGF-α, EGF and HB-EGF were 0.5 pg/ml, 7.1 pg/ml, 3.9 pg/ml and 20 pg/ml respectively.
